# miR-449a and CDK6 in gastric carcinoma

**DOI:** 10.3892/ol.2014.2370

**Published:** 2014-07-22

**Authors:** LI-PING LI, WEI-JING WU, DA-YONG SUN, ZI-YING XIE, YAN-CHUN MA, YA-GANG ZHAO

**Affiliations:** 1 Department of Gastroenterology, Guangzhou General Hospital of Guangzhou Military Command, Guangzhou, Guangdong 510010, P.R. China; 2 Department of Respiratory Medicine, The Second Affiliated Hospital of Fujian Medical University, Quanzhou, Fujian 362000, P.R. China

**Keywords:** microRNA-449, cyclin-dependent kinase 6 protein, gastric carcinoma, MGC-803, real-time cell analysis

## Abstract

The present study aimed to identify the association between microRNA (miR/miRNA)-449a, the cyclin-dependent kinase (CDK)6 protein and gastric carcinoma, and discuss the effect of miR-449a on the expression of the CDK6 protein. Quantitative (q)PCR and western blot analysis were used to analyze the expression of the miR-449a and the CDK6 protein in gastric carcinoma and tumor-adjacent normal tissues. The real-time cell analyzer and the DAPI staining test were used to monitor the different miR-449a levels regulating the proliferation and apoptosis of the MGC-803 cell line. Immunofluorescence and western blot analyses were used to detect the expression level of the CDK6 protein in the cells of the miR-449a upregulation and downregulation groups, and a control group. A scratch test was used to study the effects of miR-449a expression on migration and invasion. It was found that the expression of miR-449a was downregulated and the expression of CDK6 protein was upregulated in gastric carcinoma tissue. The level of MGC-803 cell proliferation was decreased and the apoptosis level was increased by the upregulation of miR-449a expression, and the opposite effect was shown by the downregulation of expression. The expression of the CDK6 protein in the MGC-803 cells was downregulated by upregulating the expression of miR-449a. The distance of the scratch was shortened markedly after 12 h by downregulating the expression of miR-449a in the MGC-803 cells. The present study identified that a lower expression level of miR-449 and a higher expression level of CDK6 may contribute to the occurrence and development of gastric cancer. Furthermore, it was shown that miR-449a is able to regulate the expression of the CDK6 protein.

## Introduction

Gastric cancer is the most common malignancy that occurs in the stomach mucosa, ranking first in incidence for malignant tumors of the digestive tract. Gastric cancer is among the five most common cancers in the world and is the second most prevalent cause of cancer-related mortality (1). Although it has been shown that the incidence of gastric cancer is now following a decreasing trend, the mortality rate and incidence of gastric cancer remain high in the majority of developing countries (2). The occurrence and development of gastric cancer are known to involve the imbalanced expression of proto-oncogenes and oncogenes, which are post-transcriptionally controlled by microRNA (miR/miRNA) (3,4). Therefore, the misexpression of miRNAs may affect the development of the gastric cancer by up- or downregulating the target genes. miR-449 may affect proliferation, differentiation, apoptosis and the cell cycle by regulating those target genes directly or indirectly (5). The cyclin-dependent kinase (CDK)6 protein is a regulator of the cell cycle, which can lead to cell cycle disorders, and is closely associated with cancer occurrence and development.

In the present study the association between miR-449a, the CDK6 protein and gastric carcinoma are identified. An miR-449a expression group is also established in order to observe how miR-449a affects gastric carcinoma cells, and the ability of miR-449a to regulate the expression of the CDK6 protein is discussed.

## Materials and methods

### Subject investigated and samples

A total of 66 patients, who were diagnosed with gastric cancer in the Department of Gastroenterology of Guangzhou General Hospital of Guangzhou Military Command (Guangdong, China) between January 2011 and October 2012, were enrolled in the present study. Gastric carcinoma and tumor-adjacent normal tissue samples were obtained during the surgery for the carcinoma of the stomach, and stored at −80°C in an ultra low temperature freezer (Samsung Electronics Co., Gyeonggi-do, Korea) All patients had not received any drug or chemotherapy treatments prior to surgery, and there was no significant difference in age or gender. The study was approved by the ethics committee of Guangzhou General Hospital of Guangzhou Military Command (Guangzhou, China). Patients provided written informed consent.

### Main experimental material

Lipofectamine 2000 transfection reagent was purchased from Invitrogen Life Technologies (Carlsbad, CA, USA), while TRIzol reagent, Takara reverse transcription kit, Takara real-time PCR kit, Dulbecco’s modified Eagle’s medium (DMEM; high glucose), fetal bovine serum (FBS), trypsin-EDTA, miR-449a reverse transcription and PCR primer, U6snRNA reverse transcription and PCR primer, has-miR-449a mini and has-miR-449a inhibitor were purchased from Guangzhou RiboBio Co., Ltd. (Guangzhou, Guangdong, China). Mouse monoclonal anti-rabbit CDK6 and rabbit monoclonal anti-human β-actin rhesus antibodies were purchased from Abcam Co. (Cambridge, UK), and E-Plate 16 was obtained from Roche (Basel, Switzerland). The MGC-803 cell line was supplied by the Department of Medical Experiments, Guangzhou General Hospital of Guangzhou Military Command.

### Model construction of the differential expression of miR-449a

The MGC-803 cells were cultured using DMEM with 10% FBS. Subsequent to the resuscitation of the frozen cells, the cells were provided with a 37°C, 5% CO_2_ environment. The cells in the logarithmic phase were used for the experiments. Prior to the experiments, 5 μl Lipofectamine 2000 was blended with 10 μl has-miR-449a mimic (100 nM) to prepare the has-miR-449a minic-Lipofectamine 2000 transfection reagent intermixture (termed HML). In addition, 5 μl Lipofectamine 2000 was belended with 10 μl has-miR-449a inhibitor (100 nM) to prepare the has-miR-449a inhibitor-Lipofectamine 2000 transfection reagent intermixture (termed HIL). The MGC803 cells were inoculated in cell culture plates (6-well plates), and HML was added to establish the miR-449a upregulation group, while HIL was added to establish the miR-449a downregulation group.

### Detection of miR-449a by quantitative (q)PCR

In total, 1 μl RNA template (500–550 ng/μl), 4 μl miR-449a RT Primer (62.5 nM) and 14 μl RNase-free H_2_O (Takara Bio, Inc., Shiga, Japan) were placed into PCR tubes. The PCR was performed under the following conditions: 70°C for 10 min and 0°C for 2 min. Next, the sample was mixed, and 2.5 μl 5X PrimeScript buffer, 10μl PrimeScript RT enzyme mix, 10 μl random 6-mers (100 μM), 19 μl template and 8.5 μl RNase-free H_2_O (Takara Bio, Inc.) were placed into PCR tubes. The PCR was performed under the following conditions: Reverse transcription reaction at 37°C for 15 min; and denaturation at 85°C for 5 sec. For the qPCR, 9 μl SYBR Green Mix, 2 μl miR-449a forward primer, 2μl miR-449a reverse primer, 2 μl template cDNA and 5 μl RNase-free H_2_O (Takara Bio, Inc.) were placed into the PCR tubes and underwent qPCR under the following conditions: 40 cycles of predegenration at 95°C for 20 sec; denaturation at 95°C for 10 sec; annealing at 60°C for 20 sec; prolongation (primer as a starting point, extending along the template 5′ to 3′ direction) at 70°C for 10 sec. Melting curve analysis was performed through a temperature range of 70–95°C at a rate of 0.4°C/sec.

### Detecting the CDK6 protein by western blot analysis

The protein samples were heated at 95°C for 10 min with the sample buffer (250 mM Tris-HCl, 4% sodium dodecyl sulfate, 2% β-mercaptoethanol, 10% glycerol and 0.003% bromophenol blue) and separated by 12% sodium dodecyl sulfate polyacrylamide gel electrophoresis. The protein samples were then transferred onto a polyvinylidene difluoride membrane (Millipore, Billerica, MA, USA), which were blocked with 5% skimmed dry milk in Tris-buffered saline (TBS) with 0.1 % Tween 20 (TBS-T) for 1 h. The membranes were then incubated overnight at 4°C with primary antibody diluted in 0.3% bovine serum albumin (BSA)-TBS-T. The membranes were incubated with the primary antibodies (CDK6, 1:1,000 and β-actin, 1:400; Abcam, Cambridge, UK) at 4°C for 12 h, and then with the horseradish peroxidase-linked secondary antibodies (1:1,000, goat anti-mouse monoclonal CD151 and goat anti-rabbit monoclonal β-actin; Abcam) at 37° for 1 h. The membranes were incubated with ECL Plus reagent (Amersham Biosciences, Uppsala, Sweden) and scanned using the Storm imaging system (Amersham Biosciences). Immunoreactive products were quantified using Quantity One software (Bio-Rad, Hercules, CA, USA) by determining the optical density of the protein bands.

### Real-time cell analysis

The cells of the miR-449a upregulation, miR-449a downregulation and control groups were seeded into the E-plate and plated in 37°C incubators with 5% CO_2_. The experimental specimens were scanned, and data extraction was performed by xCELLigence real-time cell analysis (RTCA; Roche).

### 4′-6-diamidino-2-phenylindole (DAPI) staining test

The cells of the miR-449a upregulation, miR-449a downregulation and control groups were seeded into the 24-well plates and plated in 37°C incubators with 5% CO_2_. The cells were stained by 0.1% DAPI-phosphate-buffered saline (PBS) subsequent to being fixed by 4% paraformaldehyde and incubated on an agitator for 30 min. Images were captured using a inverted fluorescence microscope (Olympus IX71; Olympus, Tokyo, Japan) subsequent to being mounted using glycerin.

### Cell scratch test

The cells of the miR-449a upregulation, miR-449a downregulation and control groups were seeded into the 6-well plates and plated in 37°C incubators with 5% CO_2_, diluted at 1×10^6^/ml. A scratch was formed on a single layer of cells using ultra-high temperature sterilized toothpicks, then the cells were washed three times with PBS. Images were captured using the inverted fluorescence microscope subsequent to being mounted using glycerin.

### Immunofluorescence test

The cells that reached 60–70% confluence prior to the test were seeded into the 6-well plates in 37°C incubators with 5% CO_2_. The cells of the miR-44a upregulation and downregulation groups, as well as the control group were stained by 0.1% TritonX-100-PBS after being fixed by 4% paraformaldehyde and incubated in an agitator for 10 min at room-temperature. The cells were then blocked with 3% BSA-PBS for 30 min and incubated with the primary antibodies (CDK6, 1:10; Abcam) at 4°C for 12 h, and then incubated with the secondary antibody (1:500, polyclonal goat anti-mouse IgG-H&L; Abcam) at 37°C for 1 h. Images were captured using the inverted fluorescence microscope subsequent to being mounted using glycerin.

### Statistical analysis

Statistical analyses were performed using the SPSS 13.0 software package (SPSS, Inc., Chicago, IL, USA). Normal distributions were analyzed using the one sample Kolmogorov-Smirnov test for goodness of fit and the homogeneity test of variance was performed among the groups, suggesting homogeneity of variance (P>0.05). Differences between two groups were compared using one-way analysis of variance, while multiple comparisons were compared using Fisher’s least significant difference test. P<0.05 was considered to indicate a statistically significant difference.

## Results

### Expression of miR-449a and the CDK6 protein in the gastric carcinoma tissue

**Figure 1:**
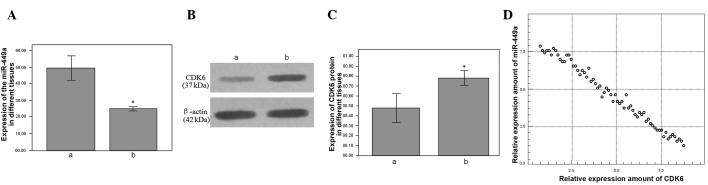
(A) Relative expression of miR-449a in gastric carcinoma and tumor-adjacent normal tissues, as determined by qPCR. ‘a’ represents the tumor-adjacent normal tissue; relative expression, 24.665±1.557. ‘b’ represents the gastric carcinoma tissue; relative expression, 49.207±13.433, P<0.001. (B) The relative expression of the CDK6 protein in the gastric carcinoma and tumor-adjacent normal tissues, as determined by western blot analysis. (C) ‘a’ represent the tumor-adjacent normal tissue; relative expression, 0.482±0.290. ‘b’ represents the gastric carcinoma tissue; relative expression, 0.792±0.165, P=0.003. (D) The relative expression of CDK6 protein was negatively correlated with the relative expression of miR-449a, as determined by Spearman’s correlation analysis, r(tumor-adjacent normal tissue)=-0.614; r(gastric carcinoma tissue)=-0.694, P<0.001. Relative expression is measured as a percentage and ^*^P<0.05 vs. tumor-adjacent normal tissues.. miR, microRNA; qPCR, quantitative PCR; CDK6, cyclin-dependent kinase 6.

The expression levels of miR-449a were compared between the gastric carcinoma and tumor-adjacent normal tissues using relative quantification, with the expression of the U6 gene as a reference. The qPCR showed that the relative expression of miR-449a was 24.665±1.557 in the gastric carcinoma tissue and 49.207±13.433 in the tumor-adjacent normal tissue, which showed that the expression of miR-449a was downregulated in the gastric carcinoma tissue (P<0.001; Fig. 1A). Western blotting was also used to test the different expression levels of the CDK6 protein in the gastric carcinoma and tumor-adjacent normal tissues; the relative expression of the CDK6 protein was upregulated in the gastric carcinoma tissue compared with the tumor-adjacent normal tissue (P<0.001; Fig. 1B and C). The relative expression of the CDK6 protein was found to be negatively associated with the relative expression of miR-449a by Spearman’s correlation analysis (Fig. 1D).

### Effects of different expression levels of miR-449a in the MGC-803 cells

**Figure 2:**
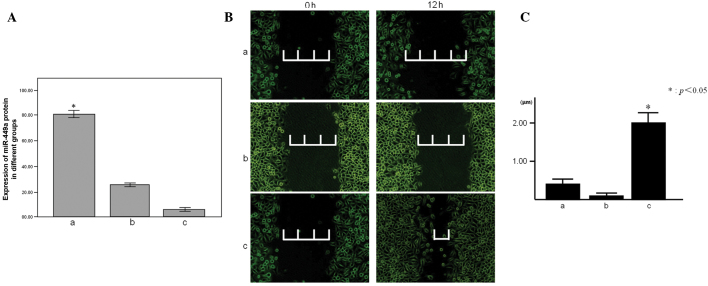
(A) Relative expression of the miR-449a. ‘a’ represents the miR-449a upregulation group; relative expression, 81.844±5.095, P<0.001. ‘b’ represents the control group; relative expression, 26.233±2.011. ‘c’ represents the miR-449a downregulation group; relative expression, 6.733±1.575. Relative expression is measured as a percentage and ^*^P<0.05. (B) The experimental result of the scratch test (x100). (C) ‘a’ represents the miR-449a upregulation group; distance change between edges of scratch, 0.503±0.080. ‘b’ represents the control group; distance change between edges of scratch, 0.198±0.068. ‘c’ represents the miR-449a downregulation group; distance change between edges of scratch, 2.519±0.350, P<0.001. The unit is of measurement is μm and ^*^P<0.05. miR, microRNA.

**Figure 3:**
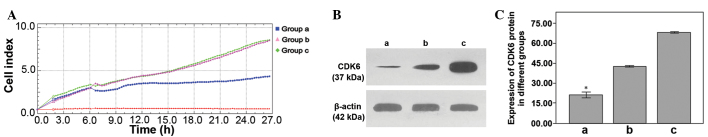
(A) Proliferation of the MGC-803 cells in 3 groups, as observed by RTCA. ‘a’ represents the miR-449a upregulation group; proliferation coefficient, 4.282±0.386, P<0.001. ‘b’ represents the control group; proliferation coefficient, 9.002±0.066. ‘c’ represents the miR-449a downregulation group; proliferation coefficient, 9.049±0.073. (B) The relative expression of the CDK6 protein in 3 groups. (C) ‘a’ represents the miR-449a upregulation group; relative expression of CDK6 protein, 21.243±4.097, P<0.001. ‘b’ represents the control group; relative expression, 41.831±1.647. ‘c’ represents the miR-449a downregulation group; relative expression, 67.418±1.369. Relative expression is measured as a percentage and ^*^P<0.05. RTCA, real-time cell analysis; miR, microRNA; CDK6, cyclin-dependent kinase 6.

**Figure 4:**
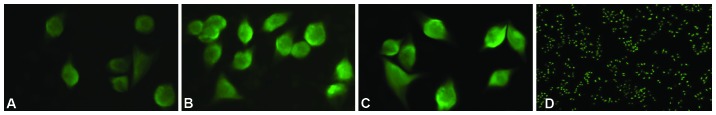
Experimental result of the immunofluorescence staining. The CDK6 protein is shown as bright green fluorescence, as observed by fluorescence microscopy using a wavelength of 475nm. (A) MGC-803 cells of the miR-449a upregulation group (magnification, ×400). (B) MGC-803 cells of the miR-449a downregulation group (magnification, ×400). (C and D) Cells of the control group. miR, microRNA; CDK6, cyclin-dependent kinase 6 (magnifications, ×400 for A and ×10 for D).

The xCELLigence RTCA system is an electronic cell sensor array that has been newly developed and is currently being tested for the dynamic monitoring of cell attachment, proliferation, damage and death (6). The expression of the miR-449a of the MGC-803 cell line was upregulated or downregulated by Lipofectamine 2000 transfection to establish the different miR-449a expression cell groups for the experiment. The qPCR result showed the different expression levels of the groups (Fig. 2A). The proliferative capacity of the MGC803 cell of the miR-449a upregulation group was shown to be decreased compared with the miR-449a downregulation and control groups by RTCA (Fig. 3A). DAPI is a useful tool that can be used to monitor the apoptosis of the cell lines, by exhibiting a strong fluorescence when DAPI becomes bound to natural double-stranded DNA; this was determined from the nuclear morphology of the MGC-803 cell line in the present experiment. The normal cell nuclei were round in shape and staining was evenly distributed. When the cell became apoptotic, the cell nuclei became deformed due to the aggregation of the DNA. The number of apoptotic cells was higher in the miR-449a upregulation group than in the downregulation and control groups (Fig. 4). The cell scratch test is a useful and straightforward way to test the migration and invasion of cells. The degree of cell migration and invasion can be determined by the mensuration of the change in distance between the edges of the scratch. In the present study, the miR-449a downregulation group was found to exhibit more cell migration and invasion than the groups, as this distance had decreased after 12 h (Fig. 2B and C).

### Effect of different expression levels of miR-449a on the expression of the CDK6 protein in the MGC-803 cells

**Figure 5:**
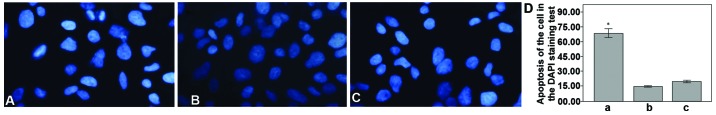
Experimental results of the DAPI staining. (A) The MGC-803 cells of the miR-449a upregulation group with nuclei stained and showing blue fluorescence (x400 magnification). (B) Cells of the miR-449a downregulation group (x400 magnification). (C) Cells of the control group (x400 magnification). (D) ‘a’ represents the MGC-803 cells of the miR-449a upregulation group; number of the apoptic cells, 68.909±7.698, P=0.002. ‘b’ represents the MGC-803 cells of the miR-449a downregulation group; number of apoptostic cells, 14.000±2.098 in 10 randomly selected fields of view. ‘c’ represents the cells of the control group; number of apoptotic cells, 21.000±3.606. ^*^P<0.001 vs. control group.

Immunofluorescence and western blot analyses are common, powerful and useful techniques that are based on the antigen-antibody reaction. In the present study, immunofluorescence and western blot analyses were used to detect the expression level of the CDK6 protein in the MGC-803 cells. It was shown that the fluorescence intensity decreased in the miR-449a upregulation group (Fig. 5) and that the relative expression of the CDK6 protein was downregulated in the miR-449a upregulation group (Fig. 3B and C).

## Discussion

More attention is currently being focused on the association between miRNA and the gastric carcinoma, in association with the pace of research on non-coding nucleic acids (3,7,8). It has been found that miR-449 is closely correlated with the occurrence and development of gastric cancer. Studies have found that miR-449 can not only induce apoptosis by affecting the E2F-p53 negative feedback loop system, but that it can also inhibit cell proliferation and cell carcinogenesis by activating the Notch signaling pathway (5). Certain studies have shown that miR-449 could directly target and switch off the expression of GMNN, MET, CCNE2 and another cancer-related genes, the majority of which are associated with the cell cycle, by syncretizing with the 3′UTR of target genes. If the miR-449 are downregulated in the gastric mucosal cell, it may ultimately lead to gastric cancer through the indefinite proliferation of the gastric mucosal cell (9). In the present study, gastric carcinoma and tumor-adjacent normal tissues were obtained from gastric cancer patients during surgery. qPCR was utilized to analyze the expression of miR-449a in the different tissues, and it was shown that the expression of miR-449a was downregulated in the gastric carcinoma tissue, which indicated that the downregulation of miR-449a was correlated with the occurrence of gastric cancer. This provide further evidence that miR-449a has a similar function to that of a cancer suppressor gene.

Cell cycle control is becoming one of the main areas of study in the field of cancer prevention. Cell cycle disorder, due to the imbalance of cell cycle element secretion, is one of the characteristics of tumor cells. CDKs and cyclins are closely associated with the development and cancerization of tissues by regulating the cell cycle, two areas that are current research hotspots (10). CDK6, a member of the CDK family, can express the CDK6 protein, which is a significant cell cycle controlling factor (11). It has been reported that the CDK6 protein is closely associated with the occurrence and development of gastric cancer (12). Cam *et al* (13) found that the expression of the CDK6 protein was maladjusted in the tumor cells. The upregulated expression of the CDK6 protein makes the G_1_ phase longer in the cells and generates a positive change in the proliferation rate. The increased cell proliferation or lessened cell apoptosis are the beginning of cancerization. The present study showed that there was downregulation of miR-449a and upregulation of the CDK6 protein in the clinical gastric cancer tissue samples. The association between the downregulation of miR-449a and the upregulation of CDK6 protein in gastric carcinoma was consequently identified, along with the association between the downregulation of miR-449a and the proliferation, apoptosis and migration of the gastric cancer MGC-803 cell line. The study showed that increased apoptosis and decreased proliferation occurred if the expression of miR-449a was upregulated in the MGC-803 cells, while cell proliferation and migration were increased the expression was downregulated. The study indicates that the expression level of miR-449a may affect the clinical pattern of cancer development gastric cancer patients.

miR-449a, which functions like a cancer suppressor gene, is closely associated with a great variety of cell cycle control genes. A previous study found that miR-449 could regulate the cell cycle-related CDK gene family (14) not only by regulating CDK-rb-e2F1 through an auto-regulatory feedback circuit (15), but also by targeting, identifying and regulating the target gene expression directly (7). Therefore, the gastric mucosal cell may have unlimited cell proliferation and finally develop gastric cancer due to the disorder of the cell cycle caused by downregulating miR-449, and miR-449a is the most common subtype of the miR-449 family (16). In the present study, the different expression levels of CDK6, affected by the different miR-449a expression levels, were analyzed using immunofluorescence and western blot analyses. The higher the expression level of miR-449a, the weaker the cell fluorescence was in the immunofluorescence analysis, and the western blotting results also showed that the expression level of miR-449a was negatively correlated with the CDK6 protein expression level. From this, it was indicated that miR-449a could regulate the expression of the CDK6 protein, and that this association may be closely correlated with the occurrence and development of gastric cancer. The abnormal expression of certain miRNAs, such as miR-196b, could indicate the occurrence of certain tumors, and miR-196b had thus been defined as a significant specific marker by scientists (17,18). As the expression of miRNA in plasma has tissue specificity, monitoring specific miRNA expression in the plasma may be a means for the early screening for cancer in high-risk groups (19).

The result of the present study have validated the fact that the downregulation of miR-449a and the upregulation of CDK6 protein participate in the occurrence and development of gastric cancer, and have also added to the data on the association between miR-449a and the CDK6 protein. We hypothesize that the correlation of gastric cancer and miRNA will become a novel direction in the future research on gastric cancer prevention, which will be good for the early diagnosis of gastric cancer by miRNA expression level screening. The present study aimed to accumulate related basic research data for cancer prevention and control through research into miR-449a, in order to improve the early diagnosis of gastric cancer patients and improve the gastric cancer survival rate.
